# Anthropogenic- and natural sources of dust in peatland during the Anthropocene

**DOI:** 10.1038/srep38731

**Published:** 2016-12-20

**Authors:** B. Fiałkiewicz-Kozieł, B. Smieja-Król, M. Frontasyeva, M. Słowiński, K. Marcisz, E. Lapshina, D. Gilbert, A. Buttler, V. E. J. Jassey, K. Kaliszan, F. Laggoun-Défarge, P. Kołaczek, M. Lamentowicz

**Affiliations:** 1 Department of Biogeography and Paleoecology, Adam Mickiewicz University, Bogumiła Krygowskiego 10, 61-680 Poznań, Poland; 2 Faculty of Earth Sciences, University of Silesia, Będzińska 60, 41-200 Sosnowiec, Poland; 3 Department of Neutron Activation Analysis, Frank Laboratory of Neutron Physics Joint Institute for Nuclear Research, Dubna, Russian Federation; 4 Department of Environmental Resources and Geohazards, Institute of Geography and Spatial Organization, Polish Academy of Sciences, Twarda 51/55, 00-818 Warszawa, Poland; 5 GFZ German Research Centre for Geosciences, Section 5.2–Climate Dynamics and Landscape Evolution, Telegrafenberg, D-14473 Potsdam, Germany; 6 Laboratory of Wetland Ecology and Monitoring, Faculty of Geographical and Geological Sciences, Adam Mickiewicz University, B.Krygowskiego 10, PL–61 680 Poznań, Poland; 7 Yugra State University, Chekhova 16, 628012, Khanty-Mansiysk, Russia; 8 Laboratoire de Chrono-environment, UMR 6249 CNRS, Université de Franche-Comté, 16 Route de Gray, 25030 Besancon Cedex, France; 9 Swiss Federal Research Institute-WSL, Community Ecology Research Unit, Station 2, CH-1015 Lausanne, Switzerland; 10 École Polytechnique Fédérale de Lausanne (EPFL), School of Architecture, Civil and Environmental, Engineering (ENAC), Laboratory of Ecological Systems (ECOS), Station 2, CH-1015 Lausanne, Switzerland; 11 Université d’Orléans, CNRS/INSU, BRGM, ISTO, UMR 7327, 45071 Orléans, France

## Abstract

As human impact have been increasing strongly over the last decades, it is crucial to distinguish human-induced dust sources from natural ones in order to define the boundary of a newly proposed epoch - the Anthropocene. Here, we track anthropogenic signatures and natural geochemical anomalies in the Mukhrino peatland, Western Siberia. Human activity was recorded there from cal AD 1958 (±6). Anthropogenic spheroidal aluminosilicates clearly identify the beginning of industrial development and are proposed as a new indicator of the Anthropocene. In cal AD 1963 (±5), greatly elevated dust deposition and an increase in REE serve to show that the geochemistry of elements in the peat can be evidence of nuclear weapon testing; such constituted an enormous force blowing soil dust into the atmosphere. Among the natural dust sources, minor signals of dryness and of the Tunguska cosmic body (TCB) impact were noted. The TCB impact was indirectly confirmed by an unusual occurrence of mullite in the peat.

Ombrotrophic peatlands are a well-known trap of atmospheric dust, trace elements and pollutants^1^,^2^,^3^,^4^ originating from both natural sources (wild fires, volcanic eruptions, dust storms, meteorite impacts)^5^,^6^,^7^,^8^ and anthropogenic sources (industry, mine, transport, nuclear tests)^1^,^9^,^10^,^11^,^12^,^13^. Knowledge about past pollution in Russia is very limited as no high-resolution peat profile of past pollution, or annual flux of trace elements and dust, has been undertaken to date.

The Siberian peatlands, one of the largest in Russia, are exceptionally interesting to explore for past geochemical signals because of their distance from any important pollution source. They are a sensitive receptor of global changes in atmospheric-dust deposition. Studies of the geochemistry of Siberian peatlands have focused predominantly on the probable impact site of the Tunguska Cosmic Body or on the pollution related to Tomsk- and Norilsk heavy industry (Fig. 1)^14^,^15^,^16^. Furthermore, no existing studies identify the recently proposed Anthropocene period, the period in which human activity dramatically altered all earth systems^17^,^18^,^19^. Here, we provide a first high-resolution record of dust flux as evidence for geochemical anomalies of both anthropogenic- and natural origin in the Mukhrino peatland in Western Siberia (Fig. 1). The Mukhrino is located over 1000 km from any large industrial center (Norilsk, Chelabinsk, Barnaul; Fig. 1). The developing oil industry in the nearest town (20 km; Khanty-Mansiysk) with a population below 100,000, seems to have a limited significance in the present context. The long distance from power plants, the ombrotrophy, the high-resolution record of palaeoecological changes all make Mukhrino a suitable place to assess human impact and the extent of airborne-particle migration – and to potentially define the boundary of the Anthropocene.

## Results and Discussion

**Figure 2:**
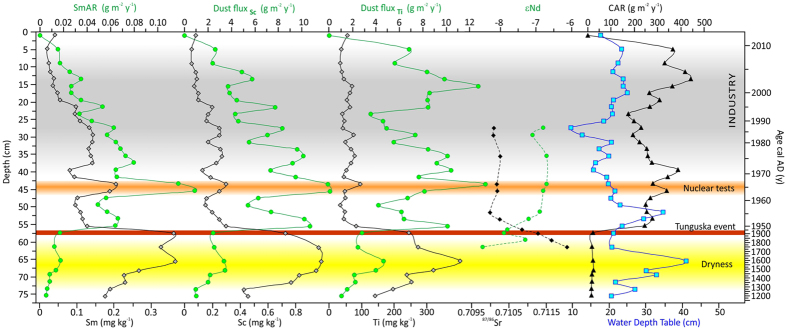
Changes in dust fluxes, carbon accumulation rate (CAR) and water depth table (DWT) during the last 800 years.

**Figure 3:**
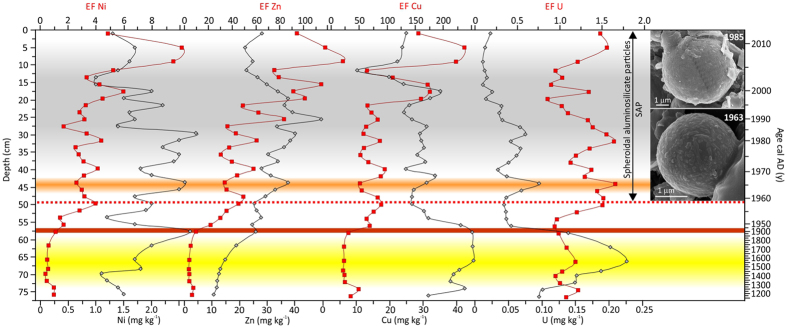
Changes in concentrations and enrichment factors of Ni, Zn, Cu and U. SEM images of technogenic spheroidal aluminosilicates (SAP). SAP are proposed as a new marker for Anthropocene; they appear only during industrial times.

The human influence on the amount and composition of atmospheric dust deposited on Mukhrino peatland can be seen to start in the late 1950 s (Figs 2 and 3). Since then, epsilon Nd values are less negative (−6.7; −7.1) compared to those from lower layers of the peat core and ^87^Sr/^86^Sr values are less radiogenic (0.70999 ± 0.000009 − 0.710269 ± 0.000017) (Table 1, Fig. 2). Less negative epsilon Nd values and lower ^87^Sr/^86^Sr values in modern Chinese dust is ascribed by Li *et al*.^20^ as due to possible addition of anthropogenic particles. Unfortunately, values of Nd and Sr isotopes of anthropogenic material from Russia are not available. Looking into shift in values of epsilon Nd and ^87^S/^86^Sr ratio after cal AD 1953 (±7) (Fig. 2) we can hypothesized, that it is the anthropogenic impact. In the layer dated cal AD 1958 (±6), the first spheroidal aluminosilicate particles (SAP) appear and remain throughout the younger part of the profile, directly confirming the addition of technogenic particles to the deposited dust (Fig. 3). This is linked with the appearance of other chronomarkers, e.g., spheroidal carbonaceous particles (SCP), whose numbers have rapidly increased everywhere after the second world war^19^. SAP are a typical inorganic, glassy component of fly ash generated during industrial coal combustion^21^,^22^,^23^,^24^,^25^ and are found even in regions located far from industrial centres, e.g., Greenland (Summit area)^26^ and the glaciers of Tianshan^27^. Ranging in size from <1 up to tens of micrometers, the majority are ideal spheres, monolithic, and dense, porous or hollow. They result from the softening, melting and vitrification of minerals such as clays, chlorite, feldspars, etc., during coal combustion in power stations^21^. Although a wide compositional variation is reported for these spherical fly-ash particles^23^, it is the SiO_2_ and Al_2_O_3_ rich varieties that are mainly encountered in peatlands; these are highly resistant to dissolution^28^,^29^. EDS spectra of individual SAP in the Mukhrino peat confirm SiO_2_ and Al_2_O_3_ as the main components, with addition of Na, K, Mg, Ti and Fe. Particles of <9 μm (average diameter 2.7 μm; n = 120) confirm a distant source for the industrial dust^28^,^30^.

The dust flux, derived from the lithogenic, conservative element scandium, varies in the peat profile from 1–12.4 g m^−2^ y^−1^ (Fig. 2). The dust deposition rate shows a dramatic increase immediately after the first SAP appearance, reaching a maximum in cal AD 1963 (±5), and falling again soon after cal AD 1966 (±5). Atmospheric weapon testing was likely the main reason for what was the highest dust flux recorded during the last 800 years, a flux unrelated to any changes in water level at that time (Fig. 2). In cal AD 1963 (±5), the maximum value of the REE accumulation rate (AR) (SmAR – 0.13 g m^−2^ y^−1^; Fig. 2) and a maximum enrichment of U (EFU – 1.65; Fig. 3) is especially notable. This finding is in line with the observed peak in concentration of La, Th and U, and increased activities of ^238^Pu and ^137^Cs, in a peat layer dated cal AD 1963 in peatlands of the Tomsk region^15^ as well with maximum of above ground nuclear tests in Russia (AD 1962)^31^. The most powerful nuclear test was Tsar Bomba detonated at Novaya Ziemlya in 1961^32^. Conversely, decreasing enrichment factors for Cu, Ni and Zn reflect the insignificance of pollution from heavy industry at that time in Mukhrino (Fig. 3). These observations further strengthen the point that nuclear tests were the reason for the first human-induced increase in dust fallout in Western Siberia. Here we show that nuclear tests not only caused the release of radionuclides, but also affected geochemical cycles of many elements, especially REE, and accelerated dust deposition.

Metals crucial for the Siberia region, i.e., Cu and Ni, exploited and processed in Norilsk and Tomsk, show constant enrichment-factor values (52–95 and 1.7–4.4, respectively; Fig. 3) in the period cal AD 1953 (±5) −1996 (±2). Small fluctuations in uranium might indicate minor influence of coal combustion or use of crude oil. A more obvious rise in the input of several metals (Ti, Cu, Ni, and Zn) is observed as late as in the year cal AD 2000 (±2) (Fig. 3). The increase of Ti dust flux at that time (Fig. 2) can be linked to the intensification of industrial activities in AD 2000. Lower enrichments of Zn, Cu in cal AD 1990 (±2) correspond to the fact that the economic system was weakened by a set of reforms during that time (Perestroika)^33^,^34^. Only in the Khanty-Mansi Autonomous District in AD 1998–2003 did emission increase by a factor of two and oil production by 37%^33^.

**Figure 4:**
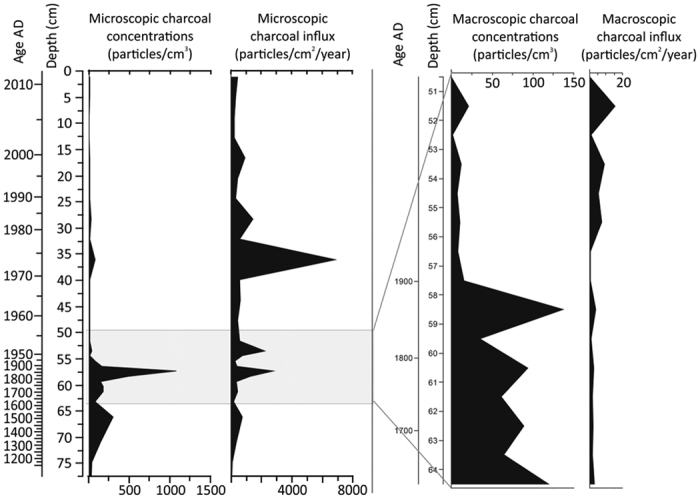
Changes of microscopic- and macroscopic charcoal concentrations and influx in the Mukhrino mire. Increased fire activity (peaks of microscopic charcoal) was probably caused by the Tunguska cosmic body event.

A depth to water table (DWT) reconstruction based on testate amoebae^35^ shows that dry conditions dominated up to cal AD 1750 (±62), with a maximum at the turn of 15^th^ and 16^th^ centuries (Fig. 2). This was followed by low carbon accumulation rates (CAR), declined signature of epsilon Nd (−8,5) and elevated ^87^Sr/^86^Sr (0.712061 ± 0.000010), similar to the signature of Chinese loess (Table 1; Sr and Nd isotopic data not available for Siberian loess). The supply of loess to dust is supported also by a characteristic value of Th/U equal 2.7–2.9, similar to the value described for loess ≈ 2.8^36^. The decline in DWT can be associated with the Little Ice Age, consistent with climatic estimations conducted for central- and eastern Europe, and with the pattern of CAR across the Northern Hemisphere and southern Siberia^37^. A slight increase in dust deposition rates (Fig. 2), and a higher charcoal content (Fig. 4) correlate with the lowest water level in the peatland, and confirm the natural inforce of dryness as a driver of dust generation.

The time of abrupt change in CAR (from 22.3–244 g m−2 y−1), dust flux and several other proxies are noted in the modelled age of the 57–58 cm layer (cal AD 1882 ± 43–1920 ± 28) and is in good agreement with the date of the Tunguska cosmic body event (TCB) which happened in June 1908 (Fig. 2) and is the best known and most mysterious extra-terrestrial event recorded in Central Russia; the amount of cosmic material dispersed into the atmosphere has been estimated at ca 1 million tons^38^,^39^.

Indirect evidence of TCB-induced dust fallout in the Mukhrino peatland is an unusual occurrence of mullite in the “Tunguska layer” and its absence in adjacent layers (Supplementary Table 2). Mullite is a high-temperature phase that forms due to the decomposition and transformation of clay minerals at temperatures >1100 °C^40^. The mineral, together with microspherules, scoria like objects (SLOs) and other high temperature minerals (corundum, suessite) lacking in the ‘Tunguska layer’, has been deemed evidence of a Younger Dryas meteorite impact^41^. In the ‘Tunguska layer’, mullite may be a product of melting of dust and soil minerals at extremely high temperatures induced by the TCB explosion. A distinct peak in microscopic charcoal contents (Fig. 4), indicative of distant fires, has been recorded in this layer together with an increased concentration of Se (Supplementary Table 1), a biogenic element released during forest fires^42^. It is known that the TCB impact set 2000 km^2^ of taiga on fire. The main stream of dust formed during the TCB explosion passed westwards through Siberia, Europe and America^7^. As to the best of our knowledge, there were no local fires during this time in the vicinity of the Mukhrino peatland, it is proposed that post TCB fires could have influenced the dust flux and the element concentration in this peat layer. The layer is characterised by the highest Th/U value (3.9; Supplementary Table 1) which would indicate a change in the supply of natural dust.

Our reconstruction of changes in the flux and composition of dust recorded in a Siberian peat profile is an important contribution to the global discussion about the boundary of the Anthropocene and the markers defining this new geological period.

The remote location of the Mukhrino peatland makes it a valuable record of natural- and anthropogenic dust sources, and especially for the differentiation of new markers of anthropogenic activity.

Prior to cal AD 1901(±37), the supply of dust was climatically driven in the main. Then, an abrupt increase of REE, a rise of microcharcoal concentrations and the occurrence of natural mullite is indicative of the influence of theTunguska Cosmic Body impact on dust influx.

The occurrence of spheroidal aluminosilicates (SAP) from cal AD 1958 (±6) onwards is clear evidence of technogenic particles in the dust supply; anthropogenic activities were, from then on, an additional source of dust. Our results show SAP occurrence in remote places and offer important evidence to recent discussion about Global Stratotype Section and Point^43^,^44^. In considerations of a Global Standard Stratigraphic Age, as spheroidal aluminosilicates are resistant in peaty- and soil environments and easy to distinguish, they may constitute, together with SCP an unambiguous global marker of the industry-induced epoch.

The influence of Siberian heavy industry on dust geochemistry has been observed as late as cal AD 2000 (±2), when economic development considerably increased after the Perestroika-related stagnancy.

Nuclear weapons tests had the main influence on REE in the Mukhrino peat profile. In Siberia, the year cal AD 1963 (±5) is marked by the highest dust input to the peat record. In addition to the release of radionuclides, the accumulation rate and enrichment of REE can be an important indicator defining nuclear tests as drivers of increased atmospheric dust.

## Methods

The Mukhrino mire is located about 20 km from Khanty-Mansiysk (60°54′N, 68°42′E) on the eastern bank of the Irtysh River in the middle taiga area of Western Siberia. A detailed site description is given in Lamentowicz *et al*.^35^ and Kremenetski *et al*.^45^. In summer 2012, a 1 m peat core was collected, sliced into 1-cm samples and divided into subsamples for various analyses. Biotic proxies (pollen analysis, macrofossil analysis, testate amoebae analysis), bulk density, ash content, charcoal and chronology, described in Lamentowicz *et al*.^35^, show the hydrological dynamics, vegetation changes and fire history of the Siberia region. In this study, we used Sr and Nd isotopes, geochemistry, mineralogy and charcoal analysis combined with the published chronology based on C^14^ age-depth model (cal AD y ± σ) and depth to water table (DWT)^35^ to reveal changes in dust flux, composition and sources.

### Element concentrations

To assess levels of pollution, 33 dry peat samples (1 g) were determined by epithermal neutron activation analysis (ENAA) for 38 main elements and REE (Supplementary Table 1). ENAA was performed at the pulsed fast reactor IBR-2 at the Frank Laboratory of Neutron Physics, JINR, Dubna, Russia. Characteristics of the neutron flux density in the two irradiation channels (one cadmium-screened) equipped with the pneumatic system and registration of gamma spectra are given elsewhere^46^. The gamma-spectra of the induced activity were processed using software developed in the Frank Laboratory of Neutron Physics^47^. Pelleted samples with masses of ca 0.3 g were heat-sealed in polyethylene foil bags for short-time irradiation and in aluminum cups for long-time irradiation. To determine medium- and long lived isotopes, namely, Na, Sc, Cr, Fe, Co, Ni, Zn, As, Se, Rb, Sr, Zr, Mo, Ag, Cd, Sb, Cs, Ba, La, Ce, Nd, Sm, Hf, Ta, W, Au, Hg, Th and U, cadmium-screened channel 1 at a resonance neutron fluency rate of 3.31 × 10^12^ n cm^−2^ s^−1^ was used. Samples were irradiated for 100 hours, repacked and, using high purity germanium detectors, measured twice after 4–5 days and 20–23 days of decay. Measurement times were 45 min and 2 hours, respectively. To determine the short-lived isotopes Mg, Al, Cl, K, Ca, Ti, V, Mn, Br, In and I, irradiation channel 2 with a thermal neutron fluency rate of 1.6 × 10^13^ n cm^−2^ s^−1^was used. Samples were irradiated for 3 min and measured twice after 3–5 min and 20 min of decay for 3 min and 9–10 min, respectively. Element contents were determined on the basis of certified reference materials and flux comparators^47^. Quality was assured by use of the Certified reference material IAEA-336 (Lichen), and NIST Standard reference materials 1632c (SRM Trace Elements in Coal (bitominous), 2710 (Highly Elevated Trace Element Concentrations (Montana Soil) and BCR (EU Community Bureau of Reference 667 (estuarine Sediment) which were irradiated simultaneously with the studied peat samples. Results were obtained with high precision and acceptable accuracy (from 3 to 15%). High precision – reproducibility of quality controls over long periods of time (years) is often better than 2% relative standard deviation (RSD).

For normalization, enrichment factors were calculated using Sc as a conservative element and reference values for the upper continental crust^48^. Element accumulation rates (g cm^−2^ y^−1^) were calculated according to the formula: element concentration* bulk density* peat accumulation rate. Dust flux (g m^−2^ y^−1^) was calculated according to the formula: (element concentration (Sc, Ti)/concentration of element in Upper Continental Crust)* bulk density *peat accumulation rate*10000^49^. Results are detailed in Supplementary Table 1.

### Mineral composition

To identify the sources of dust, the sizes, morphologies and chemical compositions of dust particles in peat samples were analyzed using Philips XL 30 ESEM and FESEM ZEISS SUPRA 35 scanning electron microscopes, both equipped with an energy dispersive system (EDS). A small portion of peat sample (~0.25 cm^3^) was air dried, gently homogenized, fixed to a carbon tab and carbon coated. Both backscattered electron (BSE) images carrying compositional information and secondary-electron (SE) images showing particle morphologies and shapes were used. BSE images enabled easy detection of all inorganic particles as they appear lighter in the dark background of peat organic matter^29^. XRD data of ashed peat samples (550 °C overnight; washed in 1 M HCl for 15 min) were obtained using a Panalytical X’Pert PRO MPD PW 3040/60 equipped with Theta-Theta geometry. Quantitative determination of crystalline phases was done by Rietveld refinement of powder diffraction data (HighScore + software). The XRD results are given in Supplementary Table 2.

### Sr and Nd isotopes

To trace the sources of atmospheric dust, ^143^Nd/^144^Nd and ^87^Sr/^86^Sr values were determined in the Isotope Laboratory at UAM. About 1 gram of peat powder was burned at 550 °C overnight. Ash was dissolved on a hot plate (~100 °C for three days) in closed PFA vials using a mixture of concentrated hydrofluoric- and nitric acids (4:1). Miniaturized chromatographic techniques described by Pin *et al*.^50^ were applied for Nd and Sr separation, using some modifications in column size and reagent concentrations introduced by Dopieralska^51^. Strontium was loaded with a TaCl_5_ activator on a single Re filament, whereas Nd were measured in a Re double-filament configuration. Sr and Nd were analyzed in dynamic collection mode on a Finnigan MAT 261 mass spectrometer.

During the course of this study, the AMES standard yielded ^143^Nd/^144^Nd = 0.512129 ± 7 (2σ mean on twenty-four analyses). The NBS 987 Sr standard gave ^87^Sr/^86^Sr of 0.710230 ± 10 (2σ mean on twenty-two analyses). ^87^Sr/^86^Sr values were normalized to ^86^Sr/^88^Sr = 0.1194 and ^143^Nd/^144^Nd values to ^146^Nd/^144^Nd = 0.7219. Total procedure blanks were less than 35 pg for Nd and Sm, and less than 80 pg for Sr. Epsilon Nd (εNd) was calculated using standard formula: εNd _sample_ = (^143/144^Nd_sample_-0,512638)/0,512638)*10000. The data are given in Table 1.

### Charcoal analysis

Microscopic charcoal was used as a proxy for regional fire activity (mainly frequency)^52^,^53^ and macroscopic charcoal to reconstruct local fires^54^,^55^. Microscopic charcoal analysis was carried out for the entire length of the profile. Samples were prepared following standard procedures for pollen analysis with addition of *Lycopodium* tablet as an indicator of concentration^56^,^57^. Microscopic charcoal particles (>10 μm) were counted as by Tinner and Hou^58^ and Finsinger and Tinner^59^. In the 50–64 cm section of the core, macroscopic charcoal analysis (particles > 100 μm; 1 cm^3^ peat samples), followed the method of Whitlock and Larsen^60^. Microscopic- and macroscopic charcoal accumulation rates (CHAR_micro_, CHAR_macro_; particles/cm^2^/yr) were calculated using the charcoal concentrations (CHAC_micro_, CHAC_macro_) and peat accumulation rates inferred from the age-depth model.

## Additional Information

**How to cite this article**: Fiałkiewicz-Kozieł, B. *et al*. Anthropogenic- and natural sources of dust in peatland during the Anthropocene. *Sci. Rep.* **6**, 38731; doi: 10.1038/srep38731 (2016).

**Publisher's note:** Springer Nature remains neutral with regard to jurisdictional claims in published maps and institutional affiliations.

## Supplementary Material

Supplementary Dataset 1

Supplementary Dataset 2

## Figures and Tables

**Figure 1:**
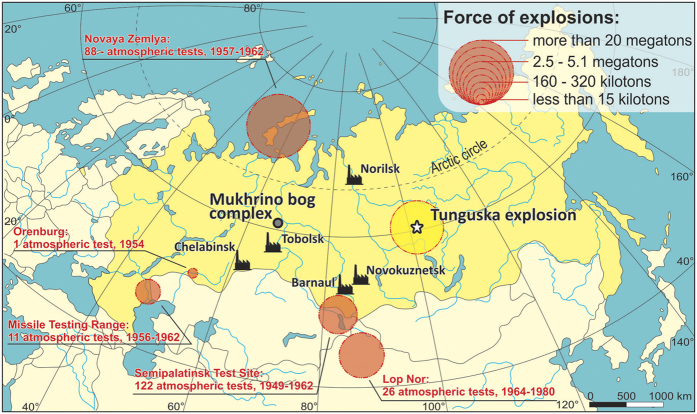
Location of the Mukhrino mire (grey circle) and the main sources of dust. The map was created using a graphical programme (Corel Draw X6, no. DR18C22VSWS5XTKC5CCB2XT8LJ7V4KH6J). Contour map previously used in Lamentowicz *et al*.^35^. Data of nuclear weapon tests were taken from Nagdy, Roser^31^. ‘Nuclear Weapons’. *Published online at OurWorldInData.org.* https://ourworldindata.org/nuclear-weapons/[Online Resource]. Factory symbol – main industrial centers in Siberia. Yellow circle with star – site of Tunguska air burst and explosion.

**Table 1:**
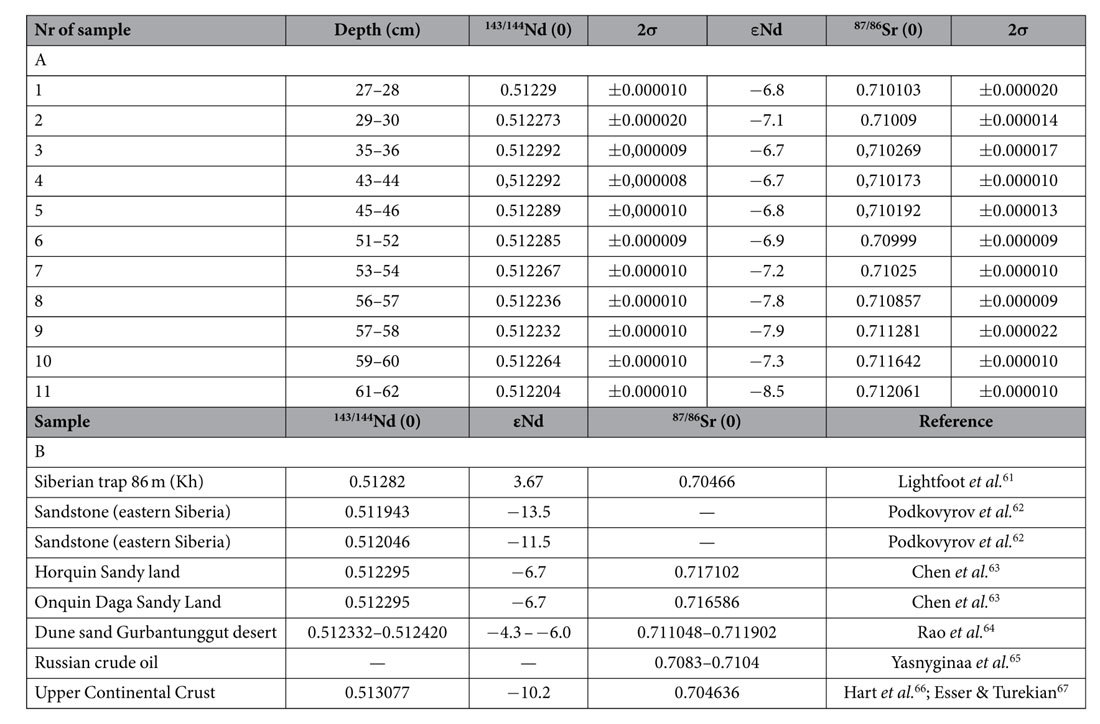
(A) Isotopic signature of peat samples from the Mukhrino peatland, (B) Examples of natural- and anthropogenic Sr and Nd signatures.
