# Supplementary material for: Anthropogenic- and natural sources of dust in peatland during the Anthropocene
Source: Sci Rep. 2016 Dec 20;6:38731. doi: 10.1038/srep38731 (PMC5171771; doi:10.1038/srep38731)
Supplement: Supplementary Dataset 2 [file srep38731-s2.doc]

Anthropogenic and natural sources of dust in peatland during the Anthropocene

Fiałkiewicz- Kozieł B., Smieja-Król B., Frontasyeva M., Słowiński M., Marcisz K., Lapshina E., Gilbert D., Buttler A., Jassey V.E.J., Kaliszan K., Laggoun-Defarge F., Kołaczek P., Lamentowicz M.

Supplementary table 2. Mineral composition (wt %) of ashed peat samples from the lower part of the profile

| Depth, cm | Mullite | Quartz | Feldspars | Illite and muscovite |
| --- | --- | --- | --- | --- |
| 44-45 | n.d. | 65.1 | 26.8 | 8.1 |
| 55-56 | n.d. | 75.2 | 22.9 | 1.8 |
| 56-57 | n.d. | 71.6 | 25.3 | 3.0 |
| 57-58 | 4.6 | 71.1 | 20.0 | 4.3 |
| 58-59 | n.d. | 75.2 | 21.8 | 3.0 |
| 59-60 | n.d. | 71.1 | 25.9 | 2.7 |
| 61-62 | n.d. | 71.8 | 25.9 | 2.1 |
| 65-66 | n.d. | 70.8 | 19.1 | 5.7 |
| 72-73 | n.d. | 68.9 | 31.3 | n.d. |

n.d. – not detected
